# Corrigendum to: Family-joining: A fast distance-based method for constructing generally labeled trees

**DOI:** 10.1093/molbev/msz281

**Published:** 2020-02-03

**Authors:** Prabhav Kalaghatgi, Nico Pfeifer, Thomas Lengauer

Mol. Biol. Evol. 33(10):2720–2734; doi:10.1093/molbev/msw123

In the original publication of this paper, there were three errors which have been corrected in the current version. On page 8, figure 4 was updated to a corrected version. On page 14, the equation on line 41 was originally listed as AIC =−2 log AIC +2m, but has been updated to AIC =−2 log-likelihood +2m. The equation on line 42 BIC =−2 log BIC +m log(n) has been corrected to BIC =−2 log-likelihood +m log(n).

