# Supplementary material for: Family-Joining: A Fast Distance-Based Method for Constructing Generally Labeled Trees
Source: Mol Biol Evol. 2016 Jul 19;33(10):2720–34. doi: 10.1093/molbev/msw123 (PMC5026249; doi:10.1093/molbev/msw123)
Supplement: Supplementary Data [file supp_33_10_2720__index.html]

Family-joining: A fast distance-based method for constructing generally labeled trees — Family-Joining: A Fast Distance-Based Method for Constructing Generally Labeled Trees — Family-Joining: A Fast Distance-Based Method for Constructing Generally Labeled Trees — Supplementary Data 

# Family-Joining: A Fast Distance-Based Method for Constructing Generally Labeled Trees

## Supplementary Data

files

- Supplementary Data - pdf file
