# Supplementary material for: Family-Joining: A Fast Distance-Based Method for Constructing Generally Labeled Trees
Source: Mol Biol Evol. 2016 Jul 19;33(10):2720–34. doi: 10.1093/molbev/msw123 (PMC5026249; doi:10.1093/molbev/msw123)
Supplement: Supplementary Data [file supp_msw123_FJ_supplement.pdf]

Supplementary material

1 Fast OLS for generally labeled trees

In what follows we show that the branch length formula, eqn. (1) (see also eqn. (10) in the main paper), that was derived by Bryant (1997) for leaf-labeled trees is also applicable for generally labeled trees. We follow the same terminology that was defined in the main paper.

Consider the internal edge e_0 shown in Fig. 1 with adjacent edges $e_1, \ldots e_k, e_{k+1} \ldots e_m$. e_0 is incident to the vertices α and β. The respective sizes of the sides of the split defined by e_0 are n_α and n_β.

For each edge e_i, define $P_i = \sum_{x \in A_i, y \in B_i} p_{xy}$ where A_i and B_i are the sides of the split defined by edge e_i. Here p_{xy} denotes the length of the path from x to y when branch lengths are determined by OLS. It turns out that $P_i = \delta_i^T d$.

For each edge e_i, $i \neq 0$, let C_i be the side of the split defined by e_i that does not contain α and β. n_i is the cardinality of C_i. Define

$$Q_i = \begin{cases} \sum_{x \in C_i} p_{\alpha x}, & \text{if } 1 \leq i \leq k \\ \sum_{x \in C_i} p_{\beta x}, & \text{if } k+1 \leq i \leq m \end{cases}$$

If both α and β are not labeled (Case 1 in Fig. 1) it can be shown that (Bryant, 1997)

$$\underline{P} = (nI - 2N)\underline{Q} + NU\underline{Q} + b_0 N \underline{v}$$

where N is the $m \times m$ diagonal matrix with $(n_1, n_2, \ldots, n_m)$ on the diagonal, I is the identity matrix, $\underline{Q} = (Q_1, Q_2, \ldots, Q_m)^T$, U is the $m \times m$ matrix of ones, $\underline{v}$ is the vector with n_β in positions 1 to k followed by n_α in positions $k+1$ to m, $\underline{P} = (P_1, P_2, \ldots, P_m)^T$, n is the total number of labeled vertices, and b_0 is the branch length of the edge e_0

Similarly for the internal edge e_0,

$$P_0 = \underline{v}^T \underline{Q} + n_\alpha n_\beta b_0$$

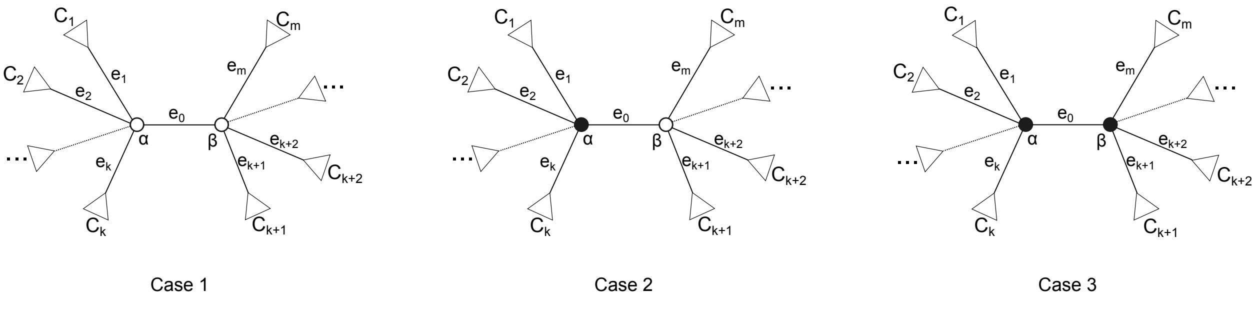

Figure 1: The three cases for the internal edge e_0. Case 1: Both α and β are not labeled. Case 2: Only α is labeled. Case 3: Both α and β are labeled. The triangles represent subtrees.

Letting $X = (nN^{-1} - 2I + U)$ and substituting $\underline{Q}$ gives the following branch length estimate.

$$b_0 = \frac{P_0 - \underline{v}^T X^{-1} N^{-1} \underline{P}}{n_\alpha n_\beta - \underline{v}^T X^{-1} \underline{v}}$$

For cases where only α and both α and β are labeled, respectively, the derivation of the equations are similar to that described in Bryant (1997) and is described below.

Case 2: α is labeled and β is not labeled

For edges e_i incident to α, $i = 1 \ldots k$, we have

$$\begin{aligned}
P_i &= \sum_{x\in A_i} \sum_{y \in B_i} p_{xy} \\
&= \sum_{j=1, j\neq i}^{m} \sum_{x\in C_i} \sum_{y\in C_j} p_{xy} + \sum_{x \in C_i} p_{\alpha x} \\
&= \sum_{j=1, j\neq i}^{k} \sum_{x\in C_i} \sum_{y\in C_j} (p_{\alpha x} + p_{\alpha y}) + \sum_{j=k+1}^{m} \sum_{x\in C_i} \sum_{y\in C_j} (p_{\alpha x} + b_0 + p_{\beta y}) + \sum_{x \in C_i} p_{\alpha x} \\
&= \sum_{j=1, j\neq i}^{k} [n_j Q_i + n_i Q_j] + \sum_{j=k+1}^{m} [n_j Q_i + n_i Q_j + n_i n_j b_0] + Q_i \\
&= (n - n_i - 1) Q_i + n_i (Q_1 + \ldots + Q_{i-1} + Q_{i+1} + \ldots + Q_m) + n_i n_\beta b_0 + Q_i \\
&= (n - 2n_i) Q_i + n_i \sum_{j=1}^{m} Q_j + n_i n_\beta b_0
\end{aligned}$$

For edges e_i incident to β, $i = k+1 \ldots m$, we have

$$\begin{aligned}
P_i &= \sum_{x\in A_i} \sum_{y \in B_i} p_{xy} \\
&= \sum_{j=1, j\neq i}^{m} \sum_{x\in C_i} \sum_{y\in C_j} p_{xy} + \sum_{x \in C_i} p_{\alpha x} \\
&= \sum_{j=1}^{k} \sum_{x\in C_i} \sum_{y\in C_j} (p_{\beta x} + b_0 + p_{\alpha y}) + \sum_{j=k+1, j\neq i}^{m} \sum_{x\in C_i} \sum_{y\in C_j} (p_{\beta x} + p_{\beta y}) + \sum_{x \in C_i} (p_{\beta x} + b_0) \\
&= (\sum_{j=1}^{k} n_j Q_i + n_i Q_j + n_i n_j b_0) + (\sum_{j=k+1, j\neq i}^{m} n_j Q_i + n_i Q_j) + Q_i + n_i b_0 \\
&= (n - n_i - 1) Q_i + n_i (Q_1 + \ldots + Q_{i-1} + Q_{i+1} + \ldots + Q_m) + n_i (n_\alpha - 1) b_0 + Q_i + n_i b_0 \\
&= (n - 2n_i) Q_i + n_i \sum_{j=1}^{m} Q_j + n_i n_\alpha b_0
\end{aligned}$$

In matrix form,

$$\begin{aligned}
&\underline{P} = (nI - 2N)\underline{Q} + NU\underline{Q} + b_0 N \underline{v} \\
&\Leftrightarrow N(nN^{-1} - 2I + U)\underline{Q} = \underline{P} - b_0 N \underline{v}
\end{aligned}$$

Setting $X = (nN^{-1} - 2I + U)$ and rearranging, we get

$$\underline{Q} = X^{-1} N^{-1} \underline{P} - b_0 X^{-1} \underline{v}$$

For the internal edge e_0 we have

$$
\begin{aligned}
P_0 &= \sum_{i=1}^{k}\sum_{j=k+1}^{m}\sum_{x\in C_i, y\in C_j} p_{xy} + \sum_{j=k+1}^{m}\sum_{x\in C_j}(b_0+p_{\beta x}) \\
&= (\sum_{i=1}^{k}\sum_{j=k+1}^{m}\sum_{x\in C_i, y\in C_j} p_{\alpha x} + b_0 + p_{\beta y}) + n_\beta b_0 + \sum_{j=k+1}^{m} Q_j \\
&= (\sum_{i=1}^{k}\sum_{j=k+1}^{m} n_j Q_i + n_i n_j b_0 + n_i Q_j) + n_\beta b_0 + \sum_{j=k+1}^{m} Q_j \\
&= \sum_{i=1}^{k} n_\beta Q_i + \sum_{j=k+1}^{m}(n_\alpha - 1)Q_j + (n_\alpha-1)n_\beta b_0 + n_\beta b_0 + \sum_{j=k+1}^{m} Q_j \\
&= \underline{v}^T\underline{Q} + n_\alpha n_\beta b_0
\end{aligned}
$$

After substituting $\underline{\mathrm{Q}}$ and rearranging we get,

$$
b_0 = \frac{P_0 - \underline{v}^T X^{-1} N^{-1} \underline{P}}{n_\alpha n_\beta - \underline{v}^T X^{-1}\underline{v}} \tag{1}
$$

Case 3: Both α and β are labeled

For edges e_i incident to α, $i = 1 \dots k$, we have

$$
\begin{aligned}
P_i &= \sum_{x\in A_i}\sum_{y\in B_i} p_{xy} \\
&= \left[\sum_{j=1, j\neq i}^{m}\sum_{x\in C_i}\sum_{y\in C_j} p_{xy}\right] + \sum_{x\in C_i} p_{\alpha x} + \sum_{x\in C_i} p_{\beta x} \\
&= \left[\sum_{j=1, j\neq i}^{k}\sum_{x\in C_i}\sum_{y\in C_j} p_{\alpha x} + p_{\alpha y}\right] + \left[\sum_{j=k+1}^{m}\sum_{x\in C_i}\sum_{y\in C_j} p_{\alpha x} + b_0 + p_{\beta y}\right] + 2\sum_{x\in C_i} p_{\alpha x} + n_i b_0 \\
&= \left[\sum_{j=1, j\neq i}^{k} n_j Q_i + n_i Q_j\right] + \left[\sum_{j=k+1}^{m} n_j Q_i + n_i Q_j + n_i n_j b_0\right] + 2Q_i + n_i b_0 \\
&= (n - n_i - 2)Q_i + n_i(Q_1 + \ldots + Q_{i-1} + Q_{i+1} + \ldots + Q_m) + n_i b_0 (1 + \sum_{j=k+1}^{m} n_j) + 2Q_i \\
&= (n - 2n_i)Q_i + n_i \sum_{j=1}^{m} Q_j + n_i n_\beta b_0
\end{aligned}
$$

By symmetry, for edges e_i incident to β, $i = k+1 \dots m$, we have,

$$
P_i = (n - 2n_i)Q_i + n_i \sum_{j=1}^{m} Q_j + n_i n_\alpha b_0
$$

In matrix form,

$$
\underline{P} = (nI - 2N)\underline{Q} + NU\underline{Q} + b_0 N \underline{v}
$$

For the internal edge e_0 we have

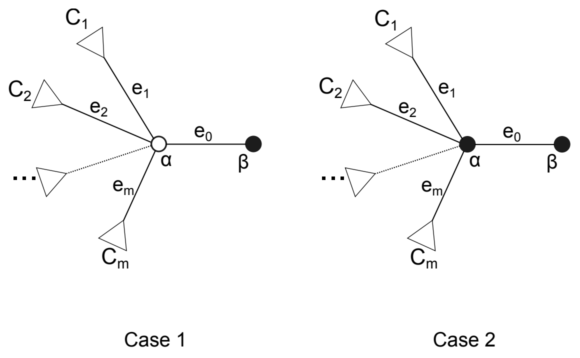

Figure 2: The two cases for the terminal edge e_0. α is labeled in case 1 and not labeled in case 2. The triangles represent subtrees.

$$
\begin{aligned}
P_0 &= \sum_{i=1}^{k}\sum_{j=k+1}^{m}\sum_{x\in C_i, y\in C_j} p_{xy} + \left[\sum_{j=1}^{k}\sum_{x\in C_j} b_0 + p_{\alpha x}\right] + \left[\sum_{j=k+1}^{m}\sum_{x\in C_j} b_0 + p_{\beta x}\right] + b_0 \\
&= \left[\sum_{i=1}^{k}\sum_{j=k+1}^{m}\sum_{x\in C_i, y\in C_j} p_{\alpha x} + b_0 + p_{\beta y}\right] + (n_\alpha + n_\beta - 1)b_0 + \sum_{j=1}^{m} Q_j \\
&= \left[\sum_{i=1}^{k}\sum_{j=k+1}^{m} n_j Q_i + n_i n_j b_0 + n_i Q_j\right] + (n_\alpha + n_\beta - 1)b_0 + \sum_{j=1}^{m} Q_j \\
&= (n_\beta - 1)\sum_{i=1}^{k} Q_i + (n_\alpha - 1)\sum_{j=k+1}^{m} Q_j + (n_\alpha - 1)(n_\beta - 1)b_0 + (n_\alpha + n_\beta - 1)b_0 + \sum_{j=1}^{m} Q_j \\
&= n_\beta \sum_{i=1}^{k} Q_i + n_\alpha \sum_{i=k+1}^{m} Q_i + n_\alpha n_\beta b_0 \\
&= \underline{v}^T \underline{Q} + n_\alpha n_\beta b_0
\end{aligned}
$$

After substituting $\underline{\mathrm{Q}}$ and rearranging we get,

$$
b_0 = \frac{P_0 - \underline{v}^T X^{-1} N^{-1} \underline{P}}{n_\alpha n_\beta - \underline{v}^T X^{-1} \underline{v}}
$$

Consider the terminal edge e_0 shown in Fig. 2 with adjacent edges $e_1, e_2 \dots e_m$. e_0 is incident to the vertices α and β. The respective sizes of the sides of the split defined by e_0 are n_α and n_β. Since e_0 is a terminal edge the leaf β is labeled. There are two cases to consider depending on if α is labeled or not labeled.

If α is not labeled (Case 1 in Fig. 2), the branch length formula given by Bryant (1997) is

$$
b_0 = \frac{P_0 - \underline{v}^T X^{-1} N^{-1} \underline{P}}{n_\alpha n_\beta - \underline{v}^T X^{-1} \underline{v}}
$$

where $n_\alpha = (n-1)$, $n_\beta = 1$ and $k = m$. If α is labeled (Case 2 in Fig. 2), the branch length formula can be derived as follows.

For edges e_i incident to α we have,

$$\begin{aligned}
P_i &= \sum_{x\in A_i}\sum_{y\in B_i} p_{xy} \\
&= \sum_{j=1,j\neq i}^{m}\sum_{x\in C_i}\sum_{y\in C_j} p_{xy} + \sum_{x\in C_i}(p_{\alpha x} + p_{\beta x}) \\
&= \sum_{j=1,j\neq i}^{m}\sum_{x\in C_i}\sum_{y\in C_j} (p_{\alpha x} + p_{\alpha y}) + \sum_{x\in C_i}(2p_{\alpha x} + b_0) \\
&= \sum_{j=1,j\neq i}^{m} [n_j Q_i + n_i Q_j] + 2Q_i + n_i b_0 \\
&= (n - n_i - 2)Q_i + n_i \sum_{j=1,j\neq i}^{m} Q_j + 2Q_i + n_i b_0 \\
&= (n - 2n_i)Q_i + n_i \sum_{j=1}^{m} Q_j + n_i b_0
\end{aligned}$$

In matrix form,

$$\underline{P} = (nI - 2N)\underline{Q} + NU\underline{Q} + b_0 N\underline{v}$$

For the terminal edge e_0 we have,

$$\begin{aligned}
P_0 &= \sum_{i=1}^{m}\sum_{x\in C_i} p_{\beta x} + b_0 \\
&= (\sum_{i=1}^{m}\sum_{x\in C_i} p_{\alpha x} + b_0) + b_0 \\
&= \sum_{i=1}^{m} Q_i + (n-1)b_0 \\
&= \underline{v}^T\underline{Q} + n_\alpha n_\beta b_0
\end{aligned}$$

where $n_\alpha = (n-1)$, $n_\beta = 1$ and $k = m$.

After substituting $\underline{\mathrm{Q}}$ and rearranging we get,

$$b_0 = \frac{P_0 - \underline{v}^T X^{-1} N^{-1} \underline{P}}{n_\alpha n_\beta - \underline{v}^T X^{-1} \underline{v}}$$

2 Molecular clock rate inferred by SA

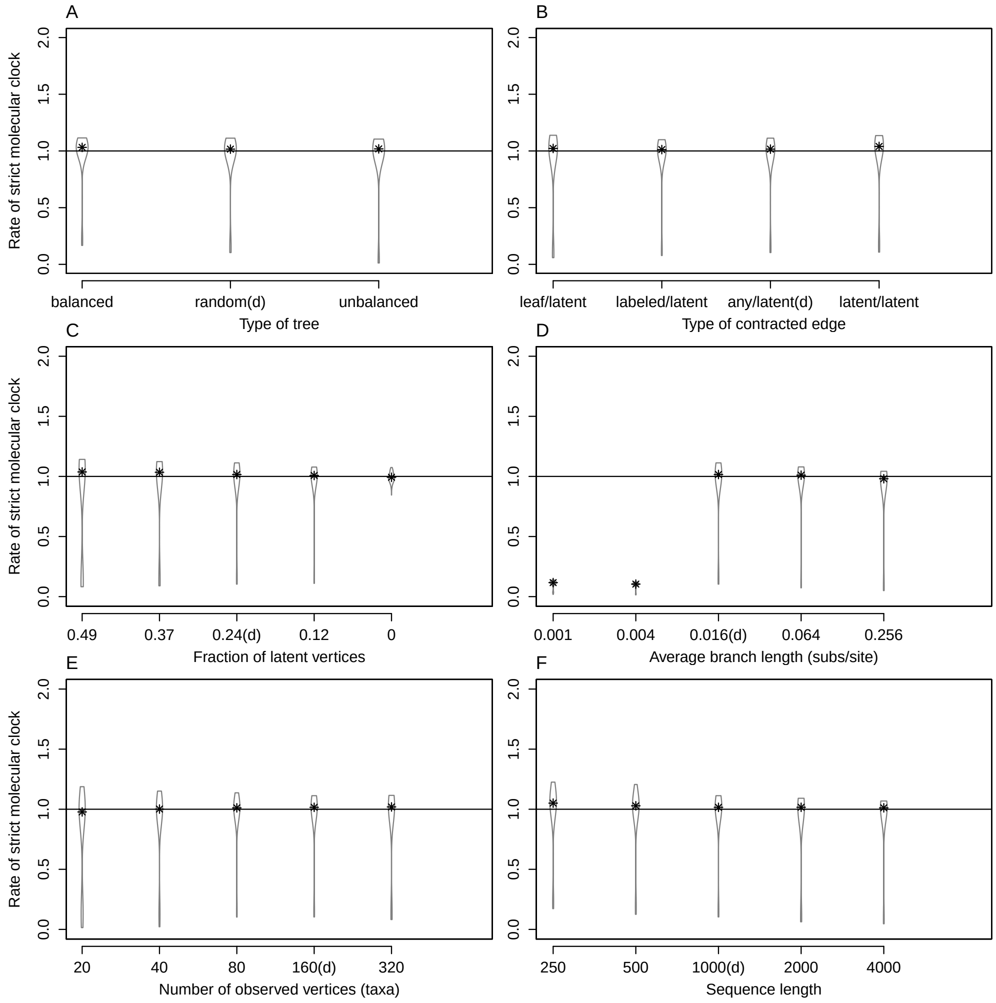

Figure 3: Rate of the strict molecular clock that is estimated by SA. The true rate of the strict molecular clock is 1.0 subs./site/time in all simulation scenarios.

3 Comparison of various FJ-based methods

For computing cross-validation error the original sequence alignment with L columns was partitioned into K validation alignments by randomly sampling L/K columns without replacement. For each validation alignment, the corresponding training alignment was constructed using the complimentary set of $L - L/K$ alignment columns. This procedure was repeated R times, giving RK training and validation alignments in total. ML distances were computed for all training and validation alignments. For a fixed value of ϵ, FJ trees were constructed for each training distance matrix. We set R to 10 and tried two values for K, i.e., 3 and 5. Test error was computed as the residual sum of squares between the fitted distances (path length on the tree) and the corresponding distances computed from the validation alignment. We then found the ϵ

that minimized expected test error as this would yield the most generalizable model.

$$\arg\min_{\epsilon} \sum_{k} \sum_{i,j} (\underbrace{d_{T(\epsilon,k)}(i,j)}_{\text{distance in fitted tree}} - \underbrace{d_{V(k)}(i,j))^2}_{\text{distance in validation set}}$$

where $T(\epsilon, k)$ is the tree constructed at threshold ϵ using distances from the kth training alignment and $V(k)$ is the kth validation alignment.

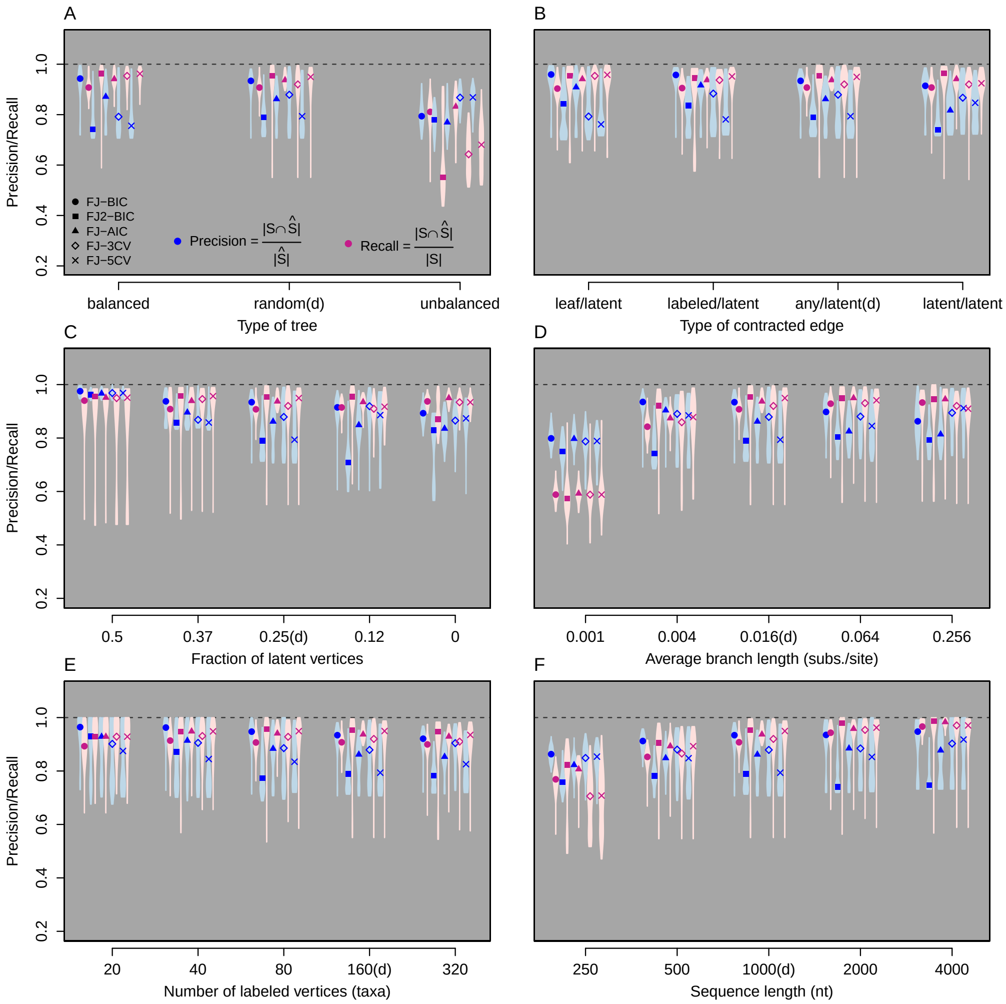

Figure 4: A comparison of various FJ-based methods. FJ-BIC is the method that is presented in the main paper. FJ2-BIC checks if siblings have a parent using the criterion shown in eqn. (4) of the main paper. FJ-AIC uses AIC for model selection. FJ-3CV and FJ-5CV performs model selection using 3-fold CV and 5-fold CV respectively.

References

Bryant, D. 1997. *Building Trees, Hunting for Trees, and Comparing Trees–Theory and method in phylogenetic analysis*. Ph.D. thesis, University of Canterbury, Christchurch, New Zealand.
